# Physiological, Immune Response, Antioxidant Capacity and Lipid Metabolism Changes in Grazing Sheep during the Cold Season

**DOI:** 10.3390/ani12182332

**Published:** 2022-09-07

**Authors:** Yanmei Zhang, Yabo Zhao, Changqing Li, Li Wang, Feng Tian, Hai Jin

**Affiliations:** 1 Inner Mongolia Key Laboratory of Animal Nutrition and Feed Science, College of Animal Science, Inner Mongolia Agricultural University, Hohhot 010018, China; 2 Inner Mongolia Academy of Agricultural & Animal Husbandry Sciences, Hohhot 010031, China

**Keywords:** Mongolian sheep *(Ovis aries)*, cold season, physiological adaptation, immune response, antioxidant capacity, lipid metabolism

## Abstract

**Simple Summary:**

As a native breed to the Inner Mongolian Plateau (Inner Mongolia, China), Mongolian sheep are tolerant to cold. However, their cold-adaptive processes, such as the physiological feedback adjustments that occur during the cold season in the plateau environment, remain unexplored. Therefore, this study aimed to evaluate the adaptations of grazing Mongolian sheep in the plateau environment by investigating the changes in physiological mechanisms and serum biochemistry of sheep reared in the warm and cold seasons. We observed that many of the biochemical functions were stimulated to meet the requirements of organismal metabolic regulation in order to enable grazing Mongolian sheep to physiologically adapt to cold climatic conditions. However, the function of resisting oxidation of the grazing Mongolian sheep was impaired during the cold season. The findings from this study provide helpful information for understanding the physiological requirements for grazing Mongolian sheep to adapt to extremely cold environments. The manuscript also provides information for optimizing the management of these animals during the cold season, increasing farm profits and designing genetic selection strategies.

**Abstract:**

Mongolian sheep are characteristically cold-tolerant. However, their cold adaptive processes, such as the physiological feedback adjustments that occur during the cold season, remain unexplored. Therefore, the present study aimed to evaluate the physiological adaptations of Mongolian sheep in cold plateau environments. A comparative analysis of the serum biochemical parameters, immune response, antioxidant capacity, and glucose and lipid metabolism of grazing Mongolian sheep in the cold and warm seasons was conducted. The results showed that in the cold season, the glucose and lipid metabolism and thermogenesis of the grazing Mongolian sheep were notably enhanced. Moreover, the immune responses were stimulated by increased levels of cytokines, such as IL-2, IL-1β, and IL-6, during the cold season. However, the antioxidant defense system was damaged; this damage was mainly characterized by decreased activity of antioxidant enzymes and an increased level of MDA during the cold season. Overall, glucose metabolism, lipid metabolism, thermogenesis, and immune responses were stimulated to meet the requirements of organismal metabolic regulation to enable grazing Mongolian sheep to physiologically adapt to cold climatic conditions.

## 1. Introduction

Extreme temperature is considered one of the main environmental stresses affecting animal production, reproduction, physiology, and health [1]. To tolerate cold exposure induced by low environmental temperatures, vertebrates have developed complex, effective, and diverse adaptation mechanisms. For example, mammals endure cold exposure via increased physical activity and food intake, improvement of their thick fur coats and insulating fat layers, or induction of the formation of brown adipose tissue and recruitment of beige adipocytes for heat production and body temperature maintenance [2,3]. More importantly, various systemic functions of organisms, including immune regulation, antioxidant defense systems, and glucose and lipid metabolism, are also stimulated appropriately to resist cold stress [4,5,6]. Guo et al. found that crossbred hybrid (small-tailed Han sheep × Hu sheep) ewes can increase the levels of serum interleukin-4 (IL-4) and the activity of various antioxidant enzymes, including catalase (CAT), superoxide dismutase (SOD), and glutathione peroxidase (GSH-Px), to resist tissue damage in a cold environment [5]. Moreover, previous studies have suggested that the plasma glucose concentration is increased to enhance energy supply in response to cold temperatures [7,8]. In addition, studies have shown that lipid metabolism-related markers, such as glycerol, total cholesterol (TCH), and triglycerides (TGs), increase during cold exposure to maintain organismal metabolism [9,10].

Mongolian sheep have the characteristics of large, fat tails; a sound physique; high-quality meat; and cold tolerance and are a traditional and important type of production animal in the Inner Mongolian Plateau [11,12,13]. The production of Mongolian sheep in most parts of the Inner Mongolian Plateau, which contains the largest grassland and natural pasture in China, is usually performed using traditional natural grazing systems, which are strongly affected by extreme cold stress. The ambient temperature in the Inner Mongolian Plateau reaches an average temperature of −14 to −28 °C during the coldest months. Despite these adverse conditions, Mongolian sheep can maintain their genetic stability well, as reported previously [14]. However, their adaptive mechanisms, such as the physiological feedback adjustments that occur during the cold season in the plateau environment, remain unknown.

Characterizing the effects of cold exposure on the physiological balance is critical for optimizing the management of grazing Mongolian sheep during the cold season, improving farm profits, and designing genetic selection strategies. Therefore, the present study aimed to assess the physiological adaptations of Mongolian sheep in the cold season by investigating the serum biochemical parameters, immune responses, antioxidant capacity, and glucose and lipid metabolism of grazing sheep reared in the warm and cold seasons.

## 2. Materials and Methods

### 2.1. Experimental Site and Climate Data

This study was conducted at the farm of Darhan Muminggan Joint Banner located in the inland arid region of Baotou (41°75′7″ N, 110°38′4″ E, 1367 m a.s.l.) on the southern Mongolian Plateau (Inner Mongolia, China) from August 2020–January 2021. Weather data were obtained from the Public Meteorological Service Center of the China Meteorological Administration (http://www.weather.com.cn/ (accessed on 1 August 2020). The climate is characterized as semiarid continental, with an annual average wind speed of 4.4 m/s, an average annual temperature of 4.2 °C, and a total annual rainfall of 257 mm. The average environmental temperatures, relative humidity, and wind velocity during the experimental period are shown in Table 1.

### 2.2. Animals, Experimental Design, and Sample Processing

A total of twenty-four Mongolian sheep ewes (10 months old, weight = 35.34 ± 2.27 kg) raised in different seasons (warm season and cold season) were used in the study. They were allocated into two groups, describing the warm (August) and cold (January) seasons, with 12 ewes assigned to each season. According to local traditional grazing management, each day the animals were herded out and grazed in the same natural pasture as the other sheep on the farm from 700 to 1900 h, and they were allowed to drink water and lick a mineral brick freely after coming back to the pen. In addition to being allowed to graze in the pasture, each ewe was fed 500 g of concentrate per day per ewe during the cold season. Samples were collected in August 2020 and January 2021. Sheep from each season were randomly selected (*n* = 6) for weight and rectal temperature assessment and slaughtered after an overnight fast; before proceeding, blood samples were collected from the animal’s jugular vein, centrifuged at 3000 r/min for 15 min (4 °C) and stored at −80 °C until they were needed for biochemical parameter analysis. Subsequently, the liver was rapidly dissected and weighed, transferred into liquid nitrogen, and further transferred into an ultralow-temperature freezer (−80 °C) for subsequent RNA extraction analysis. The tail, perirenal, large omental and small omental fat, and fur coats of the ewes reared in each season were also weighed.

Herbage samples were collected in August 2020 and January 2021 by placing five 100 × 100 cm quadrats (each quadrat was spaced over 300 m apart) in the experimental fields and cutting all of the herbage within each quadrat to ground level. The plant samples were classified, placed in an oven at 65 °C for 48 h, and then weighed to estimate the botanical composition of the forage. Then, the dry forage samples were ground through a 1 mm sieve and stored for later analysis of chemical composition. The pasture was composed of (on a dry matter basis) 34% Gramineae (mainly *Stipa* spp., *Cleistogenes* spp., and *Leymus* spp.), 38% Compositae (mainly *Artemisia* spp.), 16% Chenopodiaceae (mainly *Ceratoides* spp.), and 12% other families (mainly *Caragana Fabr.* spp. and *Allium* spp.). The ewes were fed a concentrate comprising (on a dry matter basis) 46% corn, 12% wheat bran, 12% corn bran, 10% rapeseed meal, 10% cottonseed meal, 4% soybean meal, 1.8% stone powder, 1.2% feed grade calcium hydrophosphate, 1% premix, 1% salt, and 1% carbamide. The chemical compositions of the forage grass and concentrate are shown in Table 2.

### 2.3. Determination of Serum Biochemical Parameters

The levels of high-density lipoprotein (HDL), glucose, alanine aminotransferase (ALT), and aspartate aminotransferase (AST) were measured using a Direct HDL-Cholesterol kit, glucose kit, ALT kit, and AST kit, respectively (0677, 2085, 2079, and 2080, respectively; Biosino Biotechnology Co., Ltd., Beijing, China). The levels of very-low-density lipoprotein (VLDL), immunoglobulin A (IgA), immunoglobulin M (IgM), immunoglobulin G (IgG), malondialdehyde (MDA), and total antioxidant capacity (T-AOC) and the activities of SOD, GSH-Px, glutathione (GSH), and CAT in the serum were measured in a Mindray BS-420 automatic biochemical analyzer (Shenzhen Mindray Biomedical Electronics Co. Ltd., Shenzhen, China) using a VLDL kit, IgA kit, IgM kit, IgG kit, MDA kit, T-AOC kit, SOD kit, GSH-Px kit, GSH kit, and CAT kit (HY-N0033, HY-50094, HY-50094, HY-50094, HY-60003, HY-60021, HY-M0001, HY-60005, HY-60006, and HY-60015, respectively; Beijing Sino-UK Institute of Biological Technology, Beijing, China). The concentrations of insulin, glucagon, insulin-like growth factor 1 (IGF-1), triiodothyronine (T_3_), and thyroxine (T_4_) in the serum were tested in an Enzyme Labeling Analyzer (DR-200BS; Wuxi Huawei Delang Instrument Co., Ltd., Wuxi, China) using an insulin ELISA kit, glucagon ELISA kit, IGF-1 ELISA kit, T_3_ ELISA kit, and T_4_ ELISA kit, respectively (HY-D0001, HY-10073, HY-10119, HY-400, and HY-10002, respectively; Beijing Sino-UK). The levels of interleukin-2 (IL-2), IL-4, interleukin-1β (IL-1β), and interleukin-6 (IL-6) in the serum were measured using an IL-2 ELISA kit, IL-4 ELISA kit, IL-1β ELISA kit, and IL-6 ELISA kit, respectively (H003, H005, H002, and H007, respectively; Nanjing Jiancheng Bioengineering Institute, Nanjing, China), based on the included operating instructions.

### 2.4. Total RNA Extraction, cDNA Synthesis and Quantitative Real-Time PCR (qRT-PCR)

Total RNA from each liver was isolated according to the instructions of the RNAiso Plus reagent (9109; Takara, Otsu, Japan). Final RNA samples were used to synthesize cDNA using a High-Capacity cDNA Reverse Transcription Kit (RR047A; Takara, Kusatsu City, Japan). qRT-PCR was performed using a SYBR Green Prime Script RT-PCR Kit (RR820A; Takara, Kusatsu City, Japan) on an Applied Biosystems 7500 Real Time PCR System (Bio-Rad; Berkeley, CA, USA). Primer 5.0 software was used to design primers for the genes proliferator-activated receptor alpha (*PPAR-α*), carnitine palmitoyl transferase 1B (*CPT1B*), acyl-CoA oxidase 1 (*ACOX1*), and uncoupling protein 2 (*UCP2*) [17]. The *β-actin* and *GAPDH* genes were used as the reference genes to normalize the expression of the other genes. Relative gene expression was calculated with the 2^−ΔΔCT^ algorithm [18]. The sequences of the primers and thermal conditions for qRT-PCR are listed in Table S1.

### 2.5. Statistical Analysis

All data analyses for the two seasons involved Mann–Whitney tests using SPSS version 23^®^ (International Business Machines Corporation, Armonk, NY, USA). Unless stated otherwise, the results are presented as the mean ± SEM. The statistical level of significance was set at *p* < 0.05.

## 3. Results

### 3.1. Changes in Body Weight, Rectal Temperature, and Relative Fur Coat, Fat, and Liver Weights in Grazing Mongolian Sheep

As shown in Table 3, body weight in the cold season was significantly lower than that in the warm season (*p* = 0.002). There were no significant differences in rectal temperature or in relative fur coat, fat, or liver weight between the two seasons (*p* > 0.05).

### 3.2. Changes in Serum Biochemical Parameters in Grazing Mongolian Sheep

As shown in Table 4, compared to those in the warm season, the levels of HDL, glucose, glucagon, and IGF-1 were markedly enhanced in the cold season (*p* < 0.05). However, there were no significant differences in the levels of VLDL and insulin between the cold and warm seasons (*p* > 0.05) (Table 2).

### 3.3. Changes in Lipolytic and Thermogenesis-Related Gene Expression in the Liver

**Figure 1:**
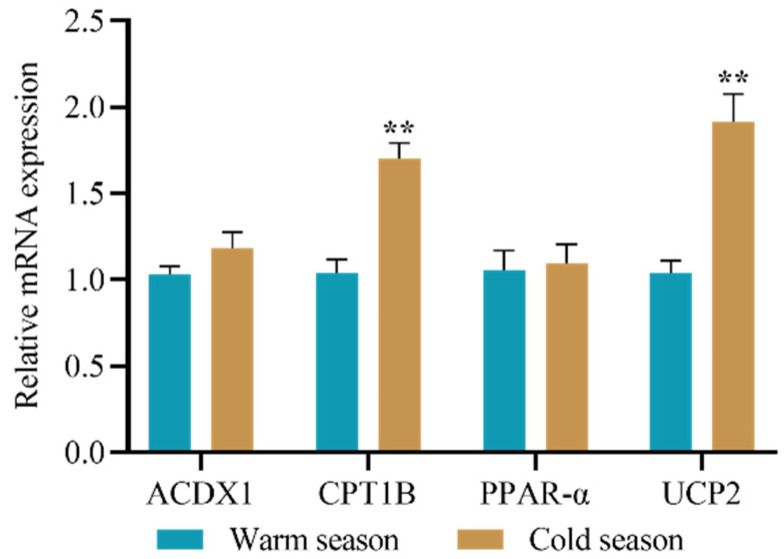
Changes in lipolytic and thermogenesis-related gene expression of livers in grazing Mongolian sheep. Each bar represents the mean ± SEM; ** indicates a *p* value < 0.01.

As shown in Figure 1, compared to that in the warm season, the mRNA expression of the thermogenesis-related gene *UCP2* and the fatty acid oxidation-related gene *CPT1B* in the liver was markedly enhanced in the cold season (*p* < 0.01, both). There were no significant differences in the expression of *ACDX1* and *PPAR-α* between the cold and warm seasons (*p* > 0.05).

### 3.4. Changes in Serum Metabolites, Hormones, and Immune-Related Parameters in Grazing Mongolian Sheep

As shown in Table 5, compared to those in the warm season, the levels of T_3_ and T_4_ were significantly decreased (all *p* < 0.01), while those of IL-2, IL-1β, and IL-6 were significantly enhanced in the cold season (*p* < 0.05, *p* < 0.01, and *p* < 0.01, respectively). However, there were no significant differences in the levels of IgA, IgG, IgM, IL-4, ALT, and AST between the two seasons (*p* > 0.05).

### 3.5. Changes in Serum Antioxidant-Related Parameters in Grazing Mongolian Sheep

As shown in Table 6, the activities of SOD and CAT and the level of T-AOC in serum in the cold season were markedly lower than those in the warm season (all *p* < 0.05). Additionally, the concentration of MDA in the cold season was significantly higher than that in the warm season (*p* < 0.01). The differences in the activity of GSH-Px and GSH between the two seasons were nonsignificant (*p* > 0.05).

## 4. Discussion

### 4.1. Changes in Body Weight, Relative Fur Coat Weight and Fat Weight in Grazing Mongolian Sheep during the Cold Season

Maintenance of a stable physiological balance in cold-exposed animals requires remarkable and complex adaptations that can drastically alter energy balances [19]. Consistent with the results of multiple studies, we found a significant reduction in body weight in grazing Mongolian sheep during the cold season, and this seasonal change may have been related to body fat mass [9,20]. However, the relative weights of the fat distributed in different parts of the sheep nonsignificantly increased and decreased during the cold season. An explanation for these results in our study could be an increase in lipid metabolism from body fat that occurred to satisfy the energy requirements of the organism during exposure to the winter environment. The lipid synthesis pathway might also have been stimulated in the cold season with the aim of maintaining a layer of fat and, consequently, insulation; however, this latter scenario may not have played as strong a role as the former. Importantly, our previous research found that the retroperitoneal and perirenal white adipose tissues of grazing Mongolian sheep undergo seasonal “browning” during the cold season [21]. The white adipose tissue “browning” process can consume stored fat for energy expenditure via nonshivering thermogenesis to generate body heat under cold exposure and can be accompanied by body weight loss [22]. In addition, the relative fur coat weight increased in the cold season, and similar findings have been reported for raccoon dogs [3]. From our data, it seems that grazing Mongolian sheep can increase the thickness of their fur coat and insulating fat as well as utilize lipid metabolism to maintain their body temperature to resist cold weather.

### 4.2. Changes in Serum Biochemical Parameters in Grazing Mongolian Sheep during the Cold Season

Some evidence has shown that plasma glucose concentrations increase to enhance energy supplies in response to cold stress [7,8]. Accordingly, Kuroshima demonstrated that the plasma glucagon and glycerol levels in rats significantly increased under cold stimulation [10]. The hormone glucagon, secreted by pancreatic alpha cells, contributes to the regulation of blood glucose via inducing hepatic glucose production in response to decreasing blood glucose [23]. Therefore, cold stress may promote energy substance secretion to maintain metabolism in organisms. Consistently, the present study showed that serum glucose and glucagon release were markedly enhanced during the cold season. These findings suggest that more energy substances are needed to meet the energy demand for physiological adaptations of grazing Mongolian sheep under cold climatic conditions than under warm conditions. IGF-1 is an important regulator of brown adipose tissue development and differentiation, which contributes to nonshivering thermogenesis [24]. In the current study, we observed that the level of IGF-1 was greatly enhanced during the cold season, indicating that thermogenic capacity was increased in grazing Mongolian sheep during the cold season. However, serum TCH, TG, and free fatty acid (FFA) levels were not significantly different between the warm and cold seasons [21], and similar findings have been reported for plateau pikas [25], suggesting that the adaptive changes in Mongolian sheep are self-compensated.

### 4.3. Changes in the mRNA Expression of Lipolytic and Thermogenesis-Related Genes in Grazing Mongolian Sheep during the Cold Season

We observed that the mRNA expression of *CPT1B* in the liver during the cold season appeared to be greater than that during the warm season, indicating that fatty acid oxidation responses are needed for the survival of Mongolian sheep in the cold season. Consistent with our findings, Sun demonstrated that the mRNA expression of *CPT1* in grouper is significantly induced by cold stress [26]. Furthermore, elevated mRNA levels of the thermogenesis-related gene *UCP2* have been observed in their livers in the cold season, which may be partially due to changes in leptin secretion. Studies have shown that leptin is involved in thermoregulation processes [27,28]. A previous study in our laboratory showed that leptin and glycerol levels were significantly increased in the cold season [21], which is consistent with research on other mammals [29]. Collectively, these findings suggest that grazing Mongolian sheep partially depend on increasing lipolytic response and thermogenesis to adapt to extremely cold environments.

### 4.4. Changes in Serum Metabolites, Hormones, and Immune-Related Parameters in Grazing Mongolian Sheep during the Cold Season

We did not detect significant differences in the serum concentrations of AST and ALT, indicating the absence of actual liver injury. Hormones, particularly those from the adrenal and thyroid glands, play a critical role in thermoregulation and physiological metabolic adjustments in sheep [30]. In the present study, the concentrations of T_3_ and T_4_ were significantly lower in the cold season than in the warm season; these results are in agreement with the findings of Ashutosh, who also reported similar changes in the concentrations of T_3_ and T_4_ in sheep during the winter [31]. One possible interpretation of these results is that the response of the thyroid to cold exposure may involve inhibition of thyroidal hormone secretion by vasoconstriction, leading to reduced production of T_3_ and T_4_. Serum IgG, IgA, and IgM production was not significantly different in grazing Mongolian sheep between the warm and cold seasons, and similar findings have been reported for humans [32].

Cytokines have marked effects on the bidirectional communication between the neuroendocrine and immune systems, and there are several proinflammatory cytokines, such as IL-1β and IL-6, which are generally considered to be indicators of inflammation that reflect the immunity of the host [32,33]. Human data have demonstrated that cold stress can increase IL-2 cytokine levels [32]. Similarly, in our study, the IL-2 level was much greater in the cold season than in the warm season. Some research has indicated that the levels of IL-1β and IL-6 are also significantly enhanced in animal models after cold stress [33,34,35]. Consistent with these findings, our data demonstrated that the concentrations of IL-1β and IL-6 in the cold season were markedly higher than those in the warm season, indicating that the immune response in grazing Mongolian sheep was stimulated under low-temperature exposure in the cold season. However, whether the adaptive immunity of grazing Mongolian sheep is weakened and whether the increases in the levels of these proinflammatory cytokines are compensatory responses that occur during prolonged low-temperature stress in the cold season should be studied further.

### 4.5. Changes in Serum Antioxidant-Related Parameters in Grazing Mongolian Sheep during the Cold Season

Many studies have shown that the antioxidant defense system is negatively affected by cold stress [36,37,38]. In this study, we observed that the activity of antioxidant enzymes in serum was negatively influenced by cold exposure in the cold season, resulting in significant decreases in the activity of SOD and CAT and the level of T-AOC. In addition, the MDA concentration was markedly enhanced during the cold season. These results suggest that the antioxidant defense system was damaged in grazing Mongolian sheep during the cold season. Therefore, optimized management, such as management incorporating cold mitigation facilities and antioxidant supplementation, is needed to minimize the damage caused by cold stress and to ultimately improve farm profits. The findings from this study provide useful information for understanding the physiological requirements for grazing Mongolian sheep to adapt to extremely cold environments.

## 5. Conclusions

In summary, cold exposure in the cold season can trigger a reduction in body weight, enhancements in glucose and lipid metabolism, and thermogenesis characterized by increases in the serum levels of glucose, glucagon, and IGF-1 and the mRNA expression of *CPT1B* and *UCP2*. In addition, cold exposure in the cold season can decrease the levels of serum hormones such as T_3_ and T_4_ and can stimulate an immune response, resulting in increased concentrations of cytokines such as IL-2, IL-1β, and IL-6. The activity of antioxidant enzymes in serum, including SOD, GSH-PX, and CAT, decreased in this study, while the MDA concentration significantly increased during the cold season. Overall, glucose metabolism, lipid metabolism, thermogenesis, and immune responses were stimulated to meet the requirements of organismal metabolism for the physiological adaptation of grazing Mongolian sheep under cold climatic conditions. However, the antioxidant defense system of the grazing Mongolian sheep was damaged during the cold season.

## Figures and Tables

**Table 1:** Climatological data during the warm and cold seasons.

Parameters	Warm Season (August)	Cold Season (January)
Average maximum temperature, °C	24.37 ± 0.48	−9.37 ± 1.33
Average minimum temperature, °C	14.04 ± 0.25	−21.11 ± 1.12
Average relative humidity, %	34.96 ± 1.84	11.86 ± 1.20
Average wind velocity, m/s	1.60 ± 0.30	4.40 ± 0.40

The data are presented as the mean ± SEM.

**Table 2:** Chemical compositions of the forage grass and concentrate (dry matter basis %).

Item	Warm Season	Cold Season	Concentrate
Crude protein ^1^	12.74	6.10	21.98
Crude fat	6.20	3.17	3.68
Neutral detergent fiber	56.36	69.07	31
Acid detergent fiber	31.46	42.8	13.40
Calcium	0.97	0.72	1.02
Phosphorus	0.2	0.07	0.6
Metabolizable energy (MJ/kg)	9.67	5.51	11.21

^1^ The levels of dry matter, crude protein and crude fat, and the metabolizable energy of the forage grass and concentrate were analyzed according to the method described by Zhang, 2007 [15]. The neutral detergent fiber and acid detergent fiber were analyzed according to Van Soest et al. 1991 [16]. The concentrations of calcium and phosphorus were determined by ethylenediamine tetraacetic acid disodium complexometric titration and the molybdenum yellow colorimetric method, respectively [15].

**Table 3:** Changes in body weight, rectal temperature, and relative fur coat, fat, and liver weights in grazing Mongolian sheep.

Parameter	Warm Season	Cold Season	*p* Value
Body weight, kg	37.963 ± 0.508	33.075 ± 0.098	0.002
Rectal temperature, °C	39.633 ± 0.156	39.667 ± 0.247	0.937
Relative weight, %			
Fur coat	8.350 ± 0.266	8.868 ± 0.217	0.818
Tail fat	2.965 ± 0.279	2.558 ± 0.178	0.310
Perirenal fat	0.602 ± 0.073	0.692 ± 0.065	0.485
Large omental fat	1.443 ± 0.141	1.483 ± 0.087	0.937
Small omental fat	0.192 ± 0.012	0.157 ± 0.017	0.132
Liver	1.467 ± 0.033	1.463 ± 0.037	0.937 ^1^

^1^ The results are presented as the mean ± SEM.

**Table 4:** Changes in serum biochemical parameters in grazing Mongolian sheep.

Parameter	Warm Season	Cold Season	*p* Value
HDL, mmol/L	1.174 ± 0.342	1.906 ± 0.563	0.026
VLDL, mmol/L	0.376 ± 0.118	0.471 ± 0.172	0.310
Glucose, mmol/L	3.729 ± 0.329	4.915 ± 0.195	0.032
Insulin, μIU/mL	13.763 ± 0.724	14.468 ± 0.929	0.132
Glucagon, pg/mL	81.063 ± 4.973	104.716 ± 14.367	0.002
IGF-1, ng/mL	156.037 ± 24.171	202.226 ± 25.482	0.015

The results are presented as the mean ± SEM. Abbreviations: HDL, high-density lipoprotein; VLDL, very-low-density lipoprotein; IGF-1, insulin-like growth factor 1.

**Table 5:** Changes in serum metabolites, hormones, and immune-related parameters in grazing Mongolian sheep.

Parameter	Warm Season	Cold Season	*p* Value
IgA, g/L	1.172 ± 0.062	1.098 ± 0.026	0.180
IgG, g/L	18.620 ± 0.559	16.985 ± 0.599	0.180
IgM, g/L	0.906 ± 0.010	0.878 ± 0.014	0.180
IL-2, pg/mL	223.427 ± 11.850	281.925 ± 20.815	0.041
IL-4, pg/mL	9.975 ± 0.952	7.698 ± 0.662	0.065
IL-1β, pg/ml	20.840 ± 0.807	25.645 ± 0.644	0.002
IL-6, pg/ml	119.617 ± 5.728	159.272 ± 4.594	0.002
T_3_, ng/ml	1.173 ± 0.020	0.998 ± 0.037	0.002
T_4_, ng/ml	66.341 ± 1.500	56.154 ± 0.942	0.002
ALT, U/L	27.831 ± 4.766	32.432 ± 4.387	0.240
AST, U/L	70.731 ± 8.926	80.610 ± 6.749	0.699

The results are presented as the mean ± SEM. Abbreviations: IgM, immunoglobulin M; IL-2, interleukin-2; IL-4, interleukin-4; IL-1β, interleukin-1β; IL-6, interleukin-6; T_3_, triiodothyronine; T_4_, thyroxine; ALT, alanine aminotransferase; AST, aspartate transaminase.

**Table 6:** Changes in serum antioxidant-related parameters in grazing Mongolian sheep.

Parameter	Warm Season	Cold Season	*p* Value
SOD, U/mL	64.220 ± 2.488	56.370 ± 0.769	0.002
MDA, nmol/mL	4.310 ± 0.092	6.790 ± 0.205	0.002
T-AOC, U/mL	10.536 ± 0.535	9.163 ± 0.231	0.041
GSH-Px, U/mL	383.057 ± 20.136	327.845 ± 11.191	0.065
GSH, μmol/L	7.540 ± 0.527	5.894 ± 0.557	0.093
CAT, U/mL	62.531 ± 2.723	53.477 ± 1.140	0.009

The results are presented as the mean ± SEM. Abbreviations: SOD, superoxide dismutase; MDA, malondialdehyde; T-AOC, total antioxidant capacity; GSH-Px, glutathione peroxidase; GSH, glutathione; CAT, catalase.

## Data Availability

The data presented in this study are openly available in FigShare at https://doi.org/10.6084/m9.figshare.20696242.v3 (accessed on 28 August 2022).

## Supplementary Materials

The following supporting information can be downloaded at https://www.mdpi.com/article/10.3390/ani12182332/s1: Table S1. Sequences of primers and thermal conditions for quantitative real-time PCR in the liver.
