# Supplementary material for: Physiological, Immune Response, Antioxidant Capacity and Lipid Metabolism Changes in Grazing Sheep during the Cold Season
Source: Animals (Basel). 2022 Sep 7;12(18):2332. doi: 10.3390/ani12182332 (PMC9495172; doi:10.3390/ani12182332)
Supplement: Supplementary file 1 [file animals-12-02332-s001.zip › animals-1886878-supplementary.pdf]

**Table S1.** The sequences of the primers and thermal conditions for qRT-PCR in the livers.

| Gene | Accession no. | Sequences (5'-3') | bp |
|---|---|---|---|
| *PPAR-α* | FJ200440.1 | F: CACGGAGTTCGCCAAGTCCATC | 122 |
| | | R: CTTTGTTCATCACAGAAGACAGCATCG | |
| *CPT1B* | NM_001009259.1 | F: GTCTGGGTGATGGGCATCTTCTTC | 105 |
| | | R: TCTGGTCAAGTGGCTGGTCTGG | |
| *ACOX1* | XM_015098942.2 | F: TGGCTACTTGAAGATGGACAACTATCG | 148 |
| | | R: CACGAGGAAGGACCTGATGAACAC | |
| *UCP2* | NM_001280682.1 | F: ATCTCCCAATGTCGCTCGCAATG | 129 |
| | | R: AAGGCAGAAGTAAAGTGGCAAGGG | |
| *β-actin* | NM_001009784 | F: CCCATTGAGCACGGCATT | 185 |
| | | R: GCAGGGGTGTTGAAGGTCTC | |
| *GAPDH* | AF022183 | F: TCCGTTGTGGATCTGACCTG | 156 |
| | | R: AGAGTGAGTGTCGCTGTTGAAGT | |
| Thermal conditions | | | |
| Stage | Reps | Temperatures, °C | Times, s |
| Initial step | 1 | 95 | 30 |
| Melt | 40 | 95 | 5 |
| Anneal/Extend | 40 | 60 | 34 |

Abbreviations: *UCP2* = Uncoupling protein 2; *PPAR-α* = Peroxisome proliferator-activated receptor alpha; *ACOX1* = acyl-CoA oxidase 1; *CPT1B* = carnitine palmitoyl transferase 1B; *β-actin* = beta-actin; *GAPDH* = Glyceraldehyde-3-phosphate dehydrogenase.
